# Levetiracetam-associated high–anion gap metabolic acidosis and secondary ketogenesis: a case series

**DOI:** 10.1093/ckj/sfag109

**Published:** 2026-04-06

**Authors:** Khaled Al-Baqain, Said Sharawi, Omar Khabbaz, Saif Ghishan, Yanal Ash-Shawareb, Murad Kheetan, Yousef Shweihat, Zeid Khitan

**Affiliations:** Department of Internal Medicine, Marshall University Joan C. Edwards School of Medicine, Huntington, WV, USA; Department of Internal Medicine, Marshall University Joan C. Edwards School of Medicine, Huntington, WV, USA; Department of Internal Medicine, Marshall University Joan C. Edwards School of Medicine, Huntington, WV, USA; Department of Internal Medicine, Marshall University Joan C. Edwards School of Medicine, Huntington, WV, USA; Department of Internal Medicine, Marshall University Joan C. Edwards School of Medicine, Huntington, WV, USA; Department of Internal Medicine, Marshall University Joan C. Edwards School of Medicine, Huntington, WV, USA; Department of Internal Medicine, Marshall University Joan C. Edwards School of Medicine, Huntington, WV, USA; Department of Internal Medicine, Marshall University Joan C. Edwards School of Medicine, Huntington, WV, USA

To the Editor,

High–anion gap metabolic acidosis (HAGMA) reflects accumulation of unmeasured anions and is most attributed to lactic acidosis, ketoacidosis, toxic alcohol ingestion, salicylate toxicity, and advanced kidney failure [1]. Drug-induced causes remain under-recognized in hospitalized patients. Levetiracetam (LEV), widely used in critically ill patients, is not routinely considered a cause of HAGMA despite pharmacokinetic and biochemical properties that could plausibly contribute to acid–base disturbances [2].

We report four patients in whom HAGMA developed following initiation or dose escalation of LEV and could not be explained by conventional aetiologies. Across all cases, evaluation excluded lactic acidosis, toxic alcohol exposure, salicylate toxicity, and pyroglutamic acidosis [1]. Organic acid profiling consistently demonstrated elevations in β-hydroxybutyrate and acetoacetate together with increased LEV metabolites. Three patients had concurrent renal dysfunction, suggesting impaired clearance as a contributing factor. Despite supportive care, the anion gap persisted or recurred until LEV was reduced or discontinued, after which metabolic acidosis improved or resolved. The clinical and biochemical characteristics of these patients are summarized in Table 1.

**Table 1:** Clinical and biochemical characteristics of patients with LEV-associated HAGMA.

Variable	Case 1	Case 2	Case 3	Case 4
Age (years)	56	52	43	31
Sex	Male	Male	Male	Female
LEV dosing	1500 mg TID	1500 mg BID	1500 mg BID	3 g load → 1 g BID
Peak anion gap	29	23	15	18
Peak creatinine (mg/dL)	4.5	3.7	1.05	4.1
eGFR prior to LEV administration or dose increase (ml/min/1.73 m²)	50.3	18.8	>60	40.3
eGFR at peak creatinine (mL/min/1.73 m²)	14.5	18.8	>60	14.2
eGFR at LEV withdrawal/resolution (mL/min/1.73 m²)	Required renal replacement therapy at time of LEV withdrawal	>60	>60	>60
Lactate at peak anion gap	Normal	Elevated	Normal	Normal (late)
Organic acid profile	↑ β-hydroxybutyrate, ↑ acetoacetate, ↑ LEV metabolites	Same	Same	Same
Resolution after LEV withdrawal	Yes	Yes	Yes	Yes

eGFR, estimated glomerular filtration rate; LEV, levetiracetam. eGFR was not calculated in patients receiving renal replacement therapy.

These findings support a mechanistic link between LEV metabolism and HAGMA. LEV contains an amide functional group within its pyrrolidone ring. Approximately one-quarter of administered LEV undergoes non-enzymatic hydrolysis of this amide bond to generate a carboxylic acid metabolite (ucb L057), which is primarily eliminated by the kidneys [2, 3]. At physiologic pH, this metabolite exists in its ionized (carboxylate) form and contributes directly to the pool of unmeasured anions. The proton released during the formation of this organic acid is buffered by serum bicarbonate, leading to bicarbonate consumption while the conjugate base remains in circulation, thereby widening the anion gap. Accumulation of this metabolite, particularly in the setting of reduced renal clearance or high dosing, provides a plausible explanation for the observed HAGMA and parallels other organic acid–mediated acidoses [1].

In addition, elevations in β-hydroxybutyrate and acetoacetate suggest secondary ketogenesis. Accumulation of drug-derived organic acids may impose metabolic stress, increasing acetyl-CoA beyond the oxidative capacity of the Krebs cycle and promoting ketone body production even in the absence of insulin deficiency. The concurrent elevation of ketone bodies and LEV metabolites supports a model in which LEV-induced organic acid accumulation is the primary driver of HAGMA, with ketogenesis representing a secondary adaptive response.

Pharmacokinetic variability in critically ill patients and reduced renal function likely increase exposure to both LEV and its metabolite, predisposing to this phenomenon [4]. A recent paediatric case has described a similar association [5]. Recognition of LEV-associated HAGMA may prevent unnecessary diagnostic testing and guide timely modification of antiseizure therapy.

## Data Availability

Data are available from the corresponding author upon request.
